# Safety and anterior chamber structure of evolution implantable Collamer lens implantation with short white-to-white corneal diameters

**DOI:** 10.3389/fmed.2022.928245

**Published:** 2022-08-17

**Authors:** Xun Chen, Fang Chen, Xuanqi Wang, Yilin Xu, Mingrui Cheng, Tian Han, Xiaoying Wang, Xingtao Zhou

**Affiliations:** ^1^Fudan University Eye Ear Nose and Throat Hospital, Shanghai, China; ^2^National Health Commission Key Lab of Myopia, Fudan University, Shanghai, China; ^3^Shanghai Research Center of Ophthalmology and Optometry, Shanghai, China; ^4^The First People’s Hospital of Zhaoqing, Zhaoqing, China

**Keywords:** myopia, EVO-ICL, white-to-white, anterior chamber, safety

## Abstract

**Introduction:**

To evaluate the safety and anterior chamber structure of implantation of the Evolution (EVO) implantable Collamer lens (EVO-ICL) in patients with short white-to-white (WTW) corneal diameters.

**Materials and methods:**

The study population was divided into two groups: the experimental group (34 eyes of 34 patients) with WTW corneal diameters of ≤10.6 mm and the control group (59 eyes of 59 patients) with WTW corneal diameters of >10.6 mm. The outcome measures included uncorrected distance visual acuity, corrected distance visual acuity, refractive power, intraocular pressure (IOP), anterior chamber angle, depth, volume, and vault.

**Results:**

The safety indices of the experimental and control groups were 1.17 ± 0.30 and 1.12 ± 0.14, respectively (*P* > 0.05); the effectiveness indices were 1.16 ± 0.31 and 1.07 ± 0.17, respectively (*P* > 0.05). The simulation curves of the expected and actual corrections in the experimental and control groups were y = 0.9876x – 0.0927 and y = 0.9799x + 0.0343, respectively. There were no significant differences between the IOPs and anterior chamber structures of the two groups (*P* > 0.05). The average vaults of the experimental and control groups were 395.76 ± 155.32 and 389.49 ± 135.01 μm, respectively (*P* > 0.05).

**Conclusion:**

EVO-ICL implantation in patients with short WTW corneal diameters (≤ 10.6 mm) was determined to be a safe, effective, and predictable method for correcting myopia. The changes in the anterior chamber structure were still within normal limits after the surgery, the IOP remained stable, and the ideal vault was achieved after the surgery.

## Highlights

- A short WTW diameter of less than 10.65 mm is usually not recommended by the current online calculator of sizing formulas, and it is not known if implantation of EVO-ICL is feasible in patients with short WTW corneal diameters.
  - In this study, we firstly presented the short-term clinical results after the EVO-ICL implantation for myopic patients with short WTW corneal diameters (≤ 10.6 mm).
- The haptics of the ICL were placed within the ciliary sulcus, and the patients with short WTW corneal diameters did not necessarily represent a small STS. The feasibility of EVO-ICL implantation in patients with short WTW corneal diameters should be determined using the detection of STS. Whether ICL implantation can be performed in patients with short WTW corneal diameters should be comprehensively judged according to the WTW, STS and anterior chamber structure.

## Introduction

The implantable Collamer lens (ICL, STAAR Surgical, Monrovia, CA, United States) has been demonstrated to be safe and effective for the correction of myopia, hyperopia, and astigmatism, and it has gained popularity as an alternative option for correcting some cases of medium to high myopia (1). Evolution (EVO) implantable Collamer lens (EVO-ICL) has a 360 μm central hole that promotes natural circulation of the aqueous humor, alleviates the pain and discomfort caused by preoperative laser iridotomy, and decreases the risks of cataracts, high intraocular pressure (IOP), and endothelial cell loss after ICL implantation (2–7). In 2014, EVO-ICL was approved for use in the Chinese market. Several myopic patients have benefited from the implantation, and the outcomes appear to be satisfying (8–10). However, there are a significant number of patients with short white-to-white (WTW) corneal diameters in China (11). There are doubts about the safety, effectiveness, and predictability of implanting EVO-ICL in patients with short WTW corneal diameters. With the current online calculator of sizing formulas recommended by STAAR Surgical, a short WTW diameter of less than 10.65 mm is usually not recommended, and it is not known if implantation of EVO-ICL is feasible in patients with short WTW corneal diameters, which are not rare in East Asian patients. In this study, we first aimed to investigate the efficacy, safety, predictability and anterior chamber structure of EVO-ICL implantation in patients with short WTW (≤ 10.6 mm) corneal diameters.

## Subjects and methods

### Compliance with ethics guidelines

This prospective study adhered to the Declaration of Helsinki and was approved by the Ethical Committee Review Board of the Fudan University Eye Ear Nose and Throat Hospital Hospital (2021018). All patients provided signed informed consent after a detailed explanation of the risks and potential outcomes of the implantation and study.

### Patients

The inclusion criteria were as follows: age between 20 and 45 years, spherical refraction of less than −4.00 D, astigmatism of up to −5.00 D, corrected distance visual acuity (CDVA) of 20/200 or better, stable refractive error (≤ 0.50 D of refractive error change within the past 2 years), no contact lens use for 1 week, and an ICL size of 12.1 mm.

The exclusion criteria included a history of ocular conditions other than myopia with or without astigmatism [suspicion of keratectasia, cornea or lens opacity, retinal detachment, glaucoma, macular degeneration, neuro-ophthalmic disease, a history of inflammation or trauma, any chronic systemic disease, ocular surgery, vertical sulcus to sulcus diameters (STS) < 10.5 mm, anterior chamber depth <2.4 mm, anterior chamber angle <25° and an endothelial cell count <2000 cells/mm^2^].

Patients were followed up for one-month; assessments of uncorrected distance visual acuity (UDVA), corrected distance visual acuity (CDVA), manifest refractive error, WTW diameter (IOL Master 500, ZEISS, Germany), STS diameters (UBM, AVISO V:4.0.2, Quantel Medical, France), corneal endothelial cell density (SP-3000P, Topcon Corporation, Japan), intraocular pressure (IOP, Tonemeterx-10, Canon, Japan), anterior chamber parameters (Pentacam HR, Type 70900; Oculus Optikgeräte GmbH, Wetzlar, Germany), and vaults (Pentacam HR, Type 70900; Oculus Optikgeräte GmbH, Wetzlar, Germany) were conducted.

Based on the diameters of the WTW, the study population was divided into two groups: the experimental group with WTW diameters of ≤10.6 mm and the control group with WTW diameters of >10.6 mm. All patients underwent routine preoperative examinations and satisfied the surgical indications for ICL implantation for the correction of medium to high myopia.

### EVO implantable Collamer lens

The EVO ICL (STAAR Surgical, Switzerland) is a plate-haptic single-piece intraocular lens made of Collamer. A 360 μm central hole was included to improve aqueous humor circulation, eliminating the need for preoperative laser peripheral iridotomy. The EVO ICL is a 6.00 mm wide lens and comes in 4 sizes (12.1, 12.6, 13.2 and 13.7 mm in length). Its optic zone diameter is 4.9–5.8 mm, with a spherical power range of −0.50 DS to −18.00 DS and a cylindrical power range of +0.50 DC to +6.00 DC. The diameter of all implanted EVO-ICLs in this study was 12.1 mm.

### Surgical technique

All surgeries were performed by experienced surgeons (XW and XZ). The implantation of ICL and the surgical procedures were the same as those used in our previous studies (8, 9). All the lenses in this study were all aligned the 180° Meridian ± 10°. During the surgery, a 3 mm temporal corneal incision was made at the temporal or superior corneoscleral limbus. Then, an EVO ICL was inserted into the anterior chamber with an injector cartridge after a viscoelastic surgical agent (1.7% sodium hyaluronate; Bausch & Lomb, China) was injected into the anterior chamber to maintain the anterior chamber depth. An additional viscoelastic agent was then placed on the top of the ICL, and an ICL positioning instrument was used to sweep the four haptics of the ICL beneath the iris. Subsequently, a balanced salt solution was used to irrigate the viscoelastic agent.

The postoperative prescription was as follows: topical 0.5% levofloxacin (Cravit; Santen) four times daily for 7 days, 1.0% prednisolone acetate (Pred Forte; Allergan, Irvine, CA, United States) four times daily for 4 days, topical pranoprofen (Senju, Osaka, Japan) four times daily for 14 days, and preservative-free artificial tears four times daily for 1 month.

### Statistical analysis

All statistical analyses were performed using SPSS (version 20.0; SPSS, Chicago, IL, United States). The results are expressed as mean ± standard deviation (SD). The Kolmogorov–Smirnov test was used to determine whether a variable was normally distributed. The independent sample *t*-test was used for normally distributed data and the Wilcoxon signed-rank test was used for abnormally distributed data. Statistical significance was set at *P* < 0.05.

## Results

### Patient demographics

The experimental group enrolled 34 eyes of 34 patients (3 men and 31 women, 18 ICLs and 16 Toric ICLs) with a mean age of 28.82 ± 5.37 years. The control group included 59 eyes of 59 patients (7 men and 52 women, 22 ICLs and 37 Toric ICLs) with a mean age of 28.00 ± 5.96 years. The baseline characteristics and preoperative biometric values of the patients are presented in Table 1. The WTWs of experimental group and control group were 10.52 ± 0.12 and 10.87 ± 0.05, respectively (*P* < 0.001). The horizontal STSs of both groups were 11.10 ± 0.36 and 11.20 ± 0.35, respectively (*P* > 0.05). There were no significant statistical differences between the two groups on the other preoperative parameters (*P* > 0.05).

**TABLE 1:** Distribution of preoperative characteristics.

Parameter	Experimental group	Control group	P value
N, eyes	34	59	
Age, years	28.82 ± 5.37(20∼44)	28.17 ± 6.23(20∼45)	0.610
logMAR UDVA	1.44 ± 0.35(1.00∼2.00)	1.47 ± 0.25(1.00∼2.00)	0.668
logMAR CDVA	0.06 ± 0.19(-0.08∼1.00)	0.02 ± 0.04(-0.08∼0.15)	0.296
Refractive errors (D)			
Spherical	−9.57 ± 3.28(-5.25∼-17.50)	−9.75 ± 2.85(-4.50∼-17.75)	0.770
Cylindrical	−1.05 ± 1.10(0.00∼-3.25)	−1.03 ± 0.98(0.00∼-4.50)	0.906
Spherical equivalent	−10.09 ± 3.33(-5.25∼-17.50)	−10.26 ± 2.91(-4.50∼-17.75)	0.797
WTW diameter (mm)	10.52 ± 0.12(10.1∼10.6)	10.87 ± 0.05(10.7∼10.9)	< 0.001
Horizontal STS	11.10 ± 0.36(10.37∼11.93)	11.20 ± 0.35(10.26∼12.08)	0.197
Vertical STS	11.58 ± 0.46(10.55∼12.35)	11.74 ± 0.40(10.77∼12.62)	0.123
IOP (mm Hg)	16.17 ± 2.71(11.5∼20.1)	16.42 ± 2.59(10.7∼21.2)	0.657
ACA (°)	37.42 ± 4.77(26.8∼48.7)	37.21 ± 5.05(26.8∼49.0)	0.842
ACD(mm)	3.00 ± 0.22(2.51∼3.49)	3.00 ± 0.22(2.64∼3.48)	0.970
ACV (ml)	163.15 ± 30.11(108∼242)	168.54 ± 25.55(108∼234)	0.361
ECD (cells/mm^2^)	2611.59 ± 306.37(2021∼3634)	2647.10 ± 236.54(1934∼3218)	0.534
ICL size (mm)	12.1	12.1	

N, number of eyes; UDVA, uncorrected distance visual acuity; CDVA, corrected distance visual acuity; D, diopters; WTW, horizontal white-to-white diameter; STS, sulcus to sulcus; IOP, intraocular pressure; ACA, anterior chamber angle; ACD, anterior chamber depth; ACV, anterior chamber volume; ECD, corneal endothelial cell density; ICL, implantable collamer lens. Results are expressed as means ± standard deviation (range).

### Safety and efficacy

**FIGURE 1:**
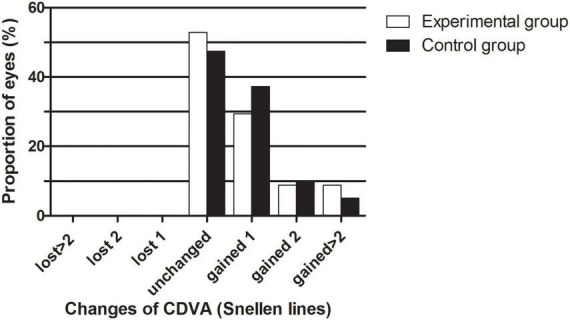
The percentage of eyes that gained/lost lines of CDVA after EVO Implantable Collamer Lens implantation between experimental and control groups.

The safety results are shown in Figure 1. The safety indices (postoperative CDVA/preoperative CDVA) of the experimental group and the control group one month after the surgeries were 1.17 ± 0.30 and 1.12 ± 0.14, respectively (*P* > 0.05). The logMAR CDVAs of the experimental group and the control group were 0.00 ± 0.13 and −0.02 ± 0.04 one month after the procedure, respectively (*P* > 0.05). In the experimental group, no eyes lost lines of CDVA, 52.94% of eyes showed no change from the baseline, 29.41% of the eyes gained one line, 8.82% of the eyes gained two lines, and 8.82% of the eyes gained two or more lines. In the control group, no eyes lost lines of CDVA, 47.46% of eyes showed no change from the baseline, 37.29% of the eyes gained one line, 10.17% of the eyes gained two lines, and 5.08% of the eyes gained two or more lines. The percentages of eyes with CDVA 20/20 or better at baseline and 1 month postoperatively were 77.14 and 88.57% in the experimental group and 66.10% and 94.92% in the control group, respectively. The percentages of eyes with a CDVA of 20/40 or better at baseline and 1 month postoperatively were 91.43 and 94.29% in the experimental group and 100.00 and 100.00% in the control group, respectively.

**FIGURE 2:**
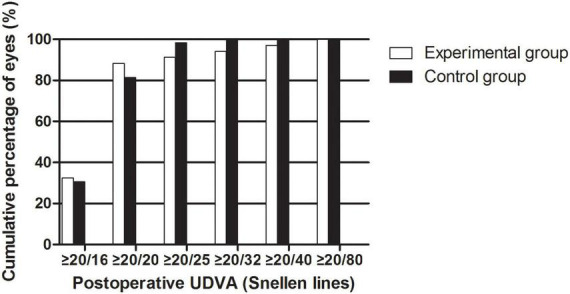
The cumulative percentage of UDVA after EVO Implantable Collamer Lens implantation between experimental and control groups.

The efficacy results are shown in Figure 2. The postoperative efficacy indices (preoperative UDVA/postoperative CDVA) of the experimental and control groups were 1.16 ± 0.31 and 1.07 ± 0.17, respectively (*P* > 0.05). The LogMAR UDVAs of the experimental group and the control group were 0.01 ± 0.14 and −0.01 ± 0.07 one month after the surgery, respectively (*P* > 0.05). The percentage of eyes with UDVAs of 20/20 or better 1 month postoperatively was 88.24% in the experimental group and 81.36% in the control group. The percentage of eyes with UDVAs of 20/40 or better 1 month postoperatively was 97.06% in the experimental group and 100.00% in the control group.

### Predictability

**FIGURE 3:**
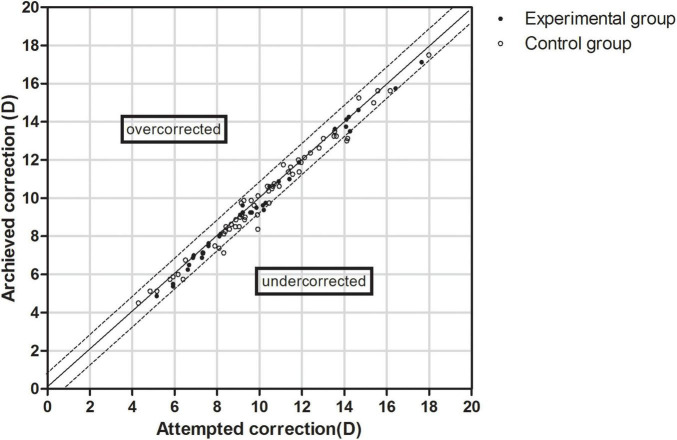
Scatter plot of attempted versus achieved correction (spherical equivalent) after EVO Implantable Collamer Lens implantation between experimental and control groups. The black solid line represented achieved correction = attempted correction, the black dotted line represented achieved correction = attempted correction ± 1.00 D.

The data of the attempted versus achieved spherical equivalent correction is shown in Figure 3. The equations of the simulation curves of the experimental and control groups were y = 0.9876x-0.0927 and y = 0.9799x + 0.0343, respectively. In the experimental group, 79.41% of the eyes were within ± 0.50 D and 100.00% were within ± 1.00 D of the expected correction. In the control group, 76.27% of the eyes were within ± 0.50 D and 93.22% were within ± 1.00 D of the expected correction (*P* > 0.05).

### Intraocular pressure and anterior chamber structure

**FIGURE 4:**
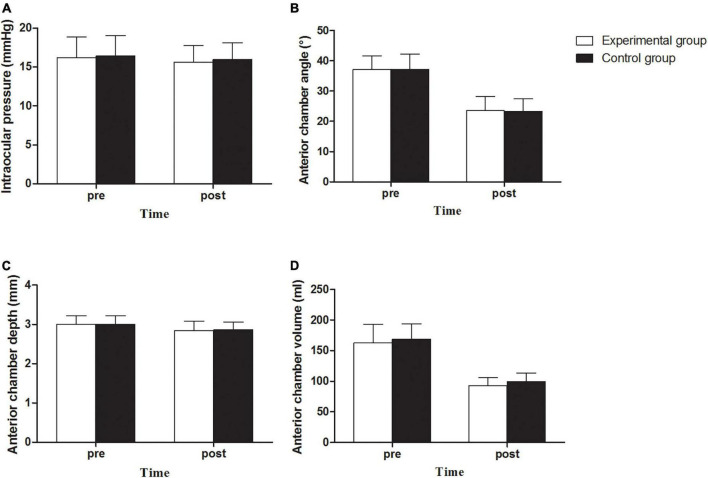
Intraocular pressure **(A)**, Anterior chamber angle **(B)**, Anterior chamber depth **(C)** and Anterior chamber volume **(D)** between experimental and control groups before and after implantation of EVO Implantable Collamer Lens.

There was no increase in IOP in the experimental and control groups. The average IOPs of the two groups were 15.64 ± 2.10 (12.0 to 19.7) and 15.94 ± 2.19 (11.6 to 21.8) mmHg (Figure 4A), respectively, showing no significant statistical difference between the two groups (*P* > 0.05) or between the preoperative and postoperative IOPs (*P* > 0.05).

There was no angle closure in the experimental and control groups. The anterior chamber angle (ACA) decreased from 37.11° ± 4.49° (26.8° to 48.7°) preoperatively to 23.58° ± 4.64° (14.1° to 37.2°) postoperatively in the experimental group, representing a decrease by 36.46% on average. A decrease from 37.21° ± 5.05° (26.8° to 49.0°) to 23.30° ± 4.23° (11.9° to 32.5°), representing a decrease by 37.37% on average, was observed in the control group. There was no statistically significant difference between the two groups (*P* > 0.05, Figure 4B). The anterior chamber depth (ACD) of the experimental group was 3.00 ± 0.22 (2.51 to 3.49) mm preoperatively and 2.84 ± 0.24 (2.16 to 3.22) mm postoperatively, with an average decrease of 5.22%; the ACD of the experimental group was 3.00 ± 0.22 (2.64 to 3.48) mm preoperatively and 2.86 ± 0.20 (2.53 to 3.33) mm postoperatively, with an average decrease of 4.83%. There was no statistically significant difference between the two groups (*P* > 0.05; Figure 4C). The anterior chamber volume (ACV) in the experimental group was 162.73 ± 30.48 (108 to 242) ml preoperatively, and it decreased to 92.64 ± 13.30 (67 to 127) ml postoperatively, with an average decrease of 43.07%. The ACV in the control group was 168.54 ± 25.55 (108 to 234) ml preoperatively, and it decreased to 99.69 ± 13.48 (76 to 137) mL postoperatively, with an average decrease of 40.85%. There was a significant difference in postoperative ACV between the two groups (*P* = 0.018, Figure 4D). There was no significant difference in the debase values of the ACA, ACD, and ACV between the two groups (*P* > 0.05).

### Vault

In the experimental group, the average postoperative vault was 395.76 ± 155.32 (170 to 780) μm, with the values for 28 eyes (82.35%) falling within the range of 250 to 750 μm. In the control group, the average postoperative vault was 389.49 ± 135.01 (180 to 760) μm, with the values of 50 eyes (84.75%) falling within the range of 250 to 750 μm. There was no statistically significant difference between the two groups (*P* > 0.05).

## Discussion

At present, there are no reports of EVO-ICL implantation in patients with short WTW corneal diameters. In this study, we firstly presented the short-term clinical results after the EVO-ICL implantation for myopic patients with short WTW corneal diameters (≤ 10.6 mm). All procedures were performed in a safe manner without adverse reactions.

In our study, the safety and efficacy indices of both groups were above 1.0, and no reduction in CDVA was found. It was safe and effective for patients with short WTW corneal diameters implanted with EVO-ICL. The results showed that the two groups had a similar predictive performance, indicating that the implantation of the EVO-ICL in patients with short WTW corneal diameters had good predictability. This was similar to the finding of a previous study involving normal WTW patient (2, 3, 12–14).

Fernandez et al. reported that after EVO-ICL implantation, the trabecular iris angle was reduced by 34.2%, 3 months after surgery in patients with normal WTW (15), which is similar to our results (37.37% reduction after the surgery). Previous studies showed that when the ACA was less than 10°, there was a risk of angle closure according to Shaffer grading system (16–18). In this study, the ACAs of the experimental and control groups were both higher than 10°. According to the reduction rate of 36.46% for the patients with short WTW corneal diameters in this study, a preoperative ACA of less than 15.74° is associated with a postoperative ACA of less than 10° and the risk of angle closure. In this study, there was no significant difference between the ACA and ACV of the experimental and control groups before surgery. At the 1-month follow-up, there was no significant difference in ACA and a significant difference in postoperative ACV between the two groups, and the ACV of the experimental group was less than that of the control group. However, there was no statistically significant difference between the debase ACV values of the two groups. In terms of the value, the preoperative ACV of the experimental group was smaller than that of the control group, and the reduction value of the ACV of the experimental group is more than that of the control group (the vault of the experimental group is higher than that of the control group, the iris moves forward more), so the postoperative ACV of the experimental group was less than that of the control group. Despite a shallower ACD, a narrower ACA, and a decreased ACV, the IOP was still within the normal range, which indicated that the changes in anterior chamber structure in patients with short WTW corneal diameters after implanting EVO-ICL were still within the safe range. The changes in anterior chamber structure caused by EVO-ICL implantation in patients with short WTW corneal diameters were similar to those in normal WTW patients, and it did not cause angle closure or IOP increase. Nevertheless, the trabecular-iris angle and the IOP should be followed closely for a longer period given that the trabecular-iris angle narrows with increased age and crystalline lens height (19).

In our study, the average vault of the experimental and control groups was lower than 500 μm because larger ICL diameters were associated with higher vaults and the implanted ICL model in this study was 12.1 mm, which was the smallest size among the ICL models (20). However, there was no difference between the two groups, and the proportions of the ideal vaults were the same, which indicated that there was no difference between patients with short WTW and ordinary WTW diameters after implanting EVO-ICL. It was feasible to implant EVO-ICL in patients with short WTW corneal diameters, and an ideal vault could also be obtained. A short WTW does not increase the probability of getting too high or too low an abnormal vault. In the experimental group, there were four eyes (11.76%) with a vault of less than 250 μm and two eye (5.82%) with a vault of more than 750 μm. Whether a low vault may cause complications, such as anterior subcapsular opacification, in these patients may require further investigation. Although two eye had a vault higher than 750 um, the IOP was still within the safe range, and the chamber angle was open. We believe that this two cases are safe and can be followed up.

In this study, although the WTW values of the experimental and control groups were statistically different before the procedure, there was no statistical difference between the STS values, indicating that the structures of the ciliary sulcus of the two groups were comparable. The haptics of the ICL were placed within the ciliary sulcus, and the patients with short WTW corneal diameters did not necessarily represent a small STS. In our previous study (21), the difference between the WTW and STS diameters was larger for the cases with an out-of-range WTW diameter or anterior chamber depth. The feasibility of EVO-ICL implantation in patients with short WTW corneal diameters should be determined using the detection of STS. Whether ICL implantation can be performed in patients with short WTW corneal diameters should be comprehensively judged according to the WTW, STS and anterior chamber structure. In our clinical experience, the values of the STS and anterior chamber structure should be adopted when the WTW ≤ 10.6 mm. When the horizontal or vertical STS ≥ 11.0 mm and the ACD ≥ 2.8 mm, the ICL could be placed in a suitable position based on the values of STS. When the horizontal and vertical STS 10.5∼11.0 mm, the ICL could be rotated to the maximum STS meridian. When the horizontal and vertical STS 10.5∼11.0 mm and the ACD 2.6∼2.8 mm, the preoperative ACA ≥ 25° is required; when the ACD 2.4∼2.6 mm, the preoperative ACA ≥ 30° is required.

There were several limitations in this study. Firstly, the patients were relatively few and the follow-up lasted for only 1 month. Complications may be observed in larger populations and over longer follow-up periods, especially for the cases with low or high vault and shallow anterior chamber. Therefore, continuous surveillance of these patients is very important to further validate the efficacy and safety of the procedure. Secondly, the cristalline lens rise, the angle to angle and the postoperative locations of the ICL haptics were not detected. Thirdly, the study had an unbalanced male-to-female ratio with more female subjects than male subjects. However, previous study (22, 23) showed that there was no significant difference in WTW diameter between men and women. Therefore, we believe that the results were not affected.

## Conclusion

In conclusion, EVO-ICL implantation in patients with a small WTW (≤ 10.6 mm) was determined to be safe, effective, and predictable for correcting myopia. The changes in the anterior chamber structure were still within normal limits post-surgery, the IOP remained stable, and the ideal vault was achieved post procedure.

## Authorship

All named authors meet the International Committee of Medical Journal Editors (ICMJE) criteria for authorship for this article, take responsibility for the integrity of the work as a whole, and have given their approval for this version to be published.

## Data availability statement

The raw data supporting the conclusions of this article will be made available by the authors, without undue reservation.

## Ethics statement

This prospective study adhered to the Declaration of Helsinki and was approved by the Ethical Committee Review Board of the Fudan University Eye Ear Nose and Throat Hospital Hospital (2021018). All patients provided signed informed consent after a detailed explanation of the risks and potential outcomes of the implantation and study. The patients/participants provided their written informed consent to participate in this study.

## Author contributions

XC, FC, XQW, YX, MC, TH, XYW, and XZ were involved in the conception or design of the work, the acquisition, analysis or interpretation of data for the work, final approval of the version to be published, and agreement to be accountable for all aspects of the work in ensuring that questions related to the accuracy or integrity of any part of the work are appropriately investigated and resolved. XC and FC were involved in the drafting the work or revising it critically for important intellectual content. All authors contributed to the article and approved the submitted version.
